# Potentiation rather than distraction in a trace fear conditioning procedure

**DOI:** 10.1016/j.beproc.2016.04.003

**Published:** 2016-07

**Authors:** M.A. Pezze, H.J. Marshall, H.J. Cassaday

**Affiliations:** School of Psychology, University of Nottingham,United Kingdom

**Keywords:** Trace conditioning, Conditioned emotional response, Attention, Distractor, Potentiation

## Abstract

Trace conditioning procedures are defined by the introduction of a trace interval between conditioned stimulus (CS, e.g. noise or light) offset and unconditioned stimulus (US, e.g. footshock). The introduction of an additional stimulus as a distractor has been suggested to increase the attentional demands of the task and to extend the usefulness of the behavioural model. In Experiment 1, the CS was noise and the distractor was provided by an intermittent light. In Experiment 2, the CS was light and the distractor was provided by an intermittent noise. In both experiments, the introduction of a 10s trace interval weakened associative learning compared with that seen in a 0s delay conditioned group. However, there was no consistent evidence of distraction. On the contrary, in Experiment 1, associative learning was stronger (in both trace and delay conditioned groups) for rats conditioned also in the presence of the intermittent light. In Experiment 2, there was no such effect when the roles of the stimuli were reversed. The results of Experiment 2 did however confirm the particular salience of the noise stimulus. The finding of increased associative learning dependent on salience is consistent with arousal-mediated effects on associative learning.

## Highlights

• Distractors should increase attentional load in trace conditioning procedures.
• Instead a light ‘distractor’ cue improved conditioning, irrespective of trace.
• This finding depended on the relative salience of the additional cue.
• There was no evidence that the distractor stimuli acquired associative strength.
• The findings are consistent with arousal-mediated effects on associative leaning.

## 1 Introduction

Trace conditioning procedures are defined by the introduction of a trace interval between conditioned stimulus (CS, e.g. noise) offset and unconditioned stimulus (US, e.g. food or footshock) onset (Kamin, 1965). The characteristic result − of reduced conditioning in consequence of temporal discontiguity − can be demonstrated in a variety of Pavlovian conditioning procedures (both appetitive and aversive) but aversive procedures have been much more widely adopted, both because acquisition is rapid and the neural circuitry necessary to basic fear conditioning is well documented.

The ability to bridge time delays to show associative learning in a trace conditioning procedure allows animals to associate what goes with what, when potentially causally-related events are separated in time. Thus, as a measure of working memory, trace conditioning holds promise as a behavioural assay for age-related memory decline: it is impaired in aged rabbits (Graves and Solomon, 1985), rats (McEchron et al., 2004, Moyer and Brown, 2006) and mice (Galvez et al., 2011, Kishimoto et al., 2001), as well as in a mouse model of senescence (Lopez-Ramos et al., 2012).

In younger adult animals, trace conditioning has been shown to require an intact hippocampus to process the temporal gap between the CS and US (McEchron et al., 1998, Weiss et al., 1999, McEchron et al., 2000, Beylin et al., 2001, Quinn et al., 2002, Rogers and Kesner, 2006) and − as is the case for tasks which measure declarative memory − seems to depend upon awareness (Clark and Squire, 1998). Consistent with known projections from hippocampus, medial prefrontal cortex (mPFC) has also been shown to be part of the trace conditioning network. Comparing across a variety of trace conditioning preparations, the emerging pattern seems to be a role for the prelimbic (PL) sub-region when memory processes are directly engaged, for example when retention is tested (Runyan et al., 2004, Oswald et al., 2008, Oswald et al., 2010), when neuronal activity is examined during a relatively long trace interval (Gilmartin and McEchron, 2005) or when longer CS durations compound the memory load (McLaughlin et al., 2002). In contrast, there is evidence to suggest that the anterior cingulate (AC) sub-region is important for earlier acquisition-related processes (Kronforst-Collins and Disterhoft, 1998, Weible et al., 2003, Weible et al., 2000, Kalmbach et al., 2009, Hattori et al., 2014). This distinction may relate to the role of AC in attentional processes and − consistent with this interpretation − excitoxic lesions of the AC sub-region of mPFC were reported to reduce trace conditioning in a mouse fear conditioning procedure which was sensitive to the effects of an experimental distractor stimulus (Han et al., 2003).

In eye-blink trace conditioning procedures, human participants’ ability to report on the CS-US relationship is similarly impaired by concurrent distraction, and this finding has also been confirmed using a trace fear conditioning procedure, in this case with a finger shock US Carter et al. (2003). This latter study was designed to be analogous to the Han et al. rodent study, though the nature of the experimental distraction was different. Carter et al. (2003) used a concurrent *n*-back task, which required participants to track previously presented digits in a list of numbers, by way of a distractor intended to compete for working memory capacity. As was the case in the Han et al. (2003) mouse conditioning study, distractor stimuli were similarly found to interfere with trace fear conditioning, delay conditioning being much more resilient to the effects of distraction (Carter et al., 2003).

Thus, it has been argued that the use of a distractor is an important procedural modification in order to model the putative attentional role of AC in a task with demonstrated sensitivity to attentional parameters and high translational relevance to our understanding of normal human ageing. Moreover, it follows that increased attentional load may be a contributing factor in the event trace conditioning deficits are demonstrated in rodent models, at least to the extent that these depend on attentional processes mediated by the AC (Pezze et al., 2016).

In a series of trace conditioning experiments using rat fear conditioning procedures, we have routinely used an extended background stimulus (Norman and Cassaday, 2003, Horsley and Cassaday, 2007, Grimond-Billa et al., 2008, Nelson et al., 2011, Pezze et al., 2016). This was provided by a continuously flashing light presented for the full duration of the conditioning session and has been intended to provide an experimental context rather than a distractor stimulus. The distractor stimulus used by Han et al. (2003) was also provided by a flashing light, different only in its temporal properties. Therefore, since distraction is of both theoretical and practical importance − to both the interpretation and demonstration of trace conditioning impairments − we adapted our existing fear conditioning procedure in an attempt to establish a reliable distractor suitable for use in rats.

It must be noted that under some experimental circumstances the introduction of extraneous stimuli is already known to result in potentiation rather than distraction (Durlach and Rescorla, 1980, Pearce et al., 1981, Rescorla, 1982, Hall and Honey, 1993). It was not our objective to add to this body of knowledge. Rather the present study sought to explore the feasibility of adapting a published distractor procedure, in order (in the longer term) to further examine the role of AC in working memory. This behavioural work was done in a rat rather than a mouse model and using a different variant of trace fear conditioning (suppression of licking rather than freezing), as per a number of earlier studies conducted to examine the neuropharmacological substrates of trace conditioning (Norman and Cassaday, 2003, Horsley and Cassaday, 2007, Grimond-Billa et al., 2008, Nelson et al., 2011, Pezze et al., 2016).

## 2 Methods

### 2.1 Animals

In each of two experiments, 48 experimentally naïve adult male Wistar rats (Charles River, UK) were caged in groups of 4 in individually ventilated cages (IVCs), on a 12:12 h light/dark cycle with food and water *ad libitum*. Cages were cleaned out twice per week and cardboard tubes and nesting materials were provided as environmental enrichment. The rats were handled for approximately 5 min per day for 1 week and then at mean weight 199 g (range 168–224 g) in Experiment 1 and 218 g (range 193–246 g) in Experiment 2 were placed on water deprivation immediately prior the conditioning procedures. One rat (in Experiment 1) was humanely killed for an unrelated reason, on the advice of the Named Veterinary Surgeon. All procedures were carried out in accordance with the United Kingdom (UK) Animals Scientific Procedures Act 1986, Project License number PPL 40/3716, which ensures full compliance with the EU Directive 2010/63/EU for animal experiments.

### 2.2 Behavioural conditioning apparatus

Four identical fully automated conditioning boxes, housed within sound-attenuating cases containing ventilation fans (Cambridge Cognition, Cambridge, UK), were used. The inner conditioning box walls consisted of plain steel (25 cm × 25 cm × 22 cm high) with a Plexiglas door (27 cm × 21 cm high), at the front. The floor was a shock grid with steel bars 1 cm apart and 1 cm above the lip of a 7 cm deep sawdust tray. A waterspout was mounted on one wall. The spout was 5 cm above the floor and connected to a lickometer supplied by a pump. Licks were registered by a break in the photobeam within the spout, which also triggered water delivery of 0.05 ml per lick. The waterspout was illuminated when water was available. A loudspeaker for the presentation of auditory stimuli was set in the roof. In Experiment 1, a 5s mixed frequency continuous noise set at 80 dB served as the CS and the distractor was an intermittent light provided by the three wall-mounted dome-shaped stimulus lights and the house light set to flash intermittently (130 ms on/off, at 8 lx, for 3 s duration with an interstimulus interval randomly chosen from 5, 10, 15 or 20 s). In Experiment 2, a 5 s flashing light served as the CS (in this case provided by the three wall mounted stimulus lights and the house light flashing (500 ms on/off, at 8 lx) and the distractor was an intermittent noise (130 ms on/off for 3 s, set at 80 dB with an interstimulus interval sequence randomly chosen from 5, 10, 15 or 20 s). Footshock of 1 s duration and 1 mA intensity provided the UCS. This was delivered through the grid floor by a constant current shock generator (pulsed voltage: output square wave 10 ms on, 80 ms off, 370 V peak under no load conditions, MISAC Systems, Newbury, UK). Stimulus control and data collection was by an Acorn Archimedes RISC computer programmed in Basic with additional interfacing using an Arachnid extension (Cambridge Cognition).

### 2.3 Behavioural conditioning procedure

Water deprivation was introduced 1 day prior to shaping and all rats received 1 h of *ad libitum* access to water in their home cage at the same time each day, in addition to access to water in the conditioning apparatus on all the experimental days except conditioning. The stages of the trace conditioning procedure were as follows:

#### 2.3.1 Pre-conditioning to establish baseline lick response

To initiate licking, rats were placed in the conditioning boxes with one of their cage mates and were shaped for 1 day until all drank from the waterspout. No data were recorded. Thereafter, animals were individually assigned to a conditioning box for the duration of the experiment (counterbalanced by experimental group). There then followed 5 days of pre-training, in which rats drank in their conditioning boxes for 15 min each day (timed from first lick). The drinking spout was illuminated throughout, but no other stimuli were presented in this phase. Latency to first lick was recorded to assess any pre-existing differences in readiness to drink (prior to conditioning).

#### 2.3.2 Conditioning with footshock

Conditioning was conducted following pre-training. No water was available within the box and the waterspout was not illuminated. There were 2 conditioning trials in which the UCS footshock was delivered at a 0 s or 10 s trace interval following termination of the 5 s CS. The first pairing of CS and UCS was presented after 5 min had elapsed, and the second pairing was 5 min after the first, followed by a further 5 min left in the apparatus. In the absence of drinking, there were no behavioural measures to record. In both experiments, the distractor was provided by an intermittent background stimulus (light in Experiment 1, noise in Experiment 2). For animals in the distractor condition, the presentation of the distractor started 1 min before the first CS-UCS pairing and ended 1 min after the final CS-UCS presentation.

#### 2.3.3 Reshaping after footshock

On the day following conditioning, animals were reshaped following the same procedure as in pre-training sessions. This was done in order to re-establish drinking after conditioning. Additionally, the reshaping latencies provided a measure of contextual conditioning as reflected in suppression to the contextual cues provided by the experimental chambers.

#### 2.3.4 Conditioned suppression tests

On the day following reshaping, the animals were placed in the conditioning boxes and underwent an extinction test to the CS. Water was available throughout the test and the waterspout was illuminated. Once the animals had made 50 licks, the CS was presented for 15 min. The latency to make 50 licks in the absence of the CS (the A period, timed from the first lick made in each box) provided a measure of any individual variation in baseline lick responding. This was compared with the time taken to complete 50 licks following CS onset (B period) in a suppression ratio (A/(A + B)) to assess the level of conditioning to the CS, adjusted for any individual variation in drink rate. Conditioned suppression was also measured as the number of licks made during the first 1 min of CS presentation. A second extinction test measured suppression to the distractor stimulus in the same way. The extinction tests were run in a counterbalanced order.

### 2.4 Experimental design and statistical analysis

In both experiments, there were 2 conditioning groups conditioned with or without a concurrently presented distractor stimulus in a 2 × 2 factorial design at levels 0 or 10 s trace interval and with or without the distractor (n = 12/group). Statistical analyses were performed using analysis of variance (ANOVA). The pre-conditioning and reshaping dependent variables were lick latencies, as well as the number of licks made in the first 1 min of the session. The test dependent variables were the suppression ratios, as well as the number of licks made during the first 1 min test presentation. The same variables were analysed in the same way for the tests of suppression to the CS and the distractor stimulus. However, examination of the effects of trace interval on suppression to the distractor was necessarily restricted to those rats in the groups actually presented with a distractor. Finally, a combined analysis compared conditioning as a function of noise versus light target CS identity and light versus noise distractor identity. Non-significant effects on baseline responding are not reported.

## 3 Results

### 3.1 Experiment 1: noise CS with intermittent light distractor

**Fig. 1:**
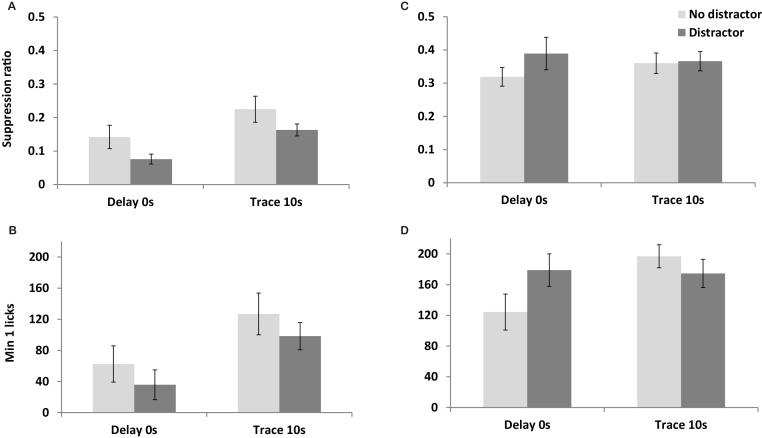
shows suppression measures of conditioning to the noise CS and light distractor in Experiment 1. Fig. 1A shows the level of conditioning to the noise CS expressed as mean suppression ratio (calculated as A/(A + B); where A was the time taken to complete 50 licks prior to CS presentation and B was the time taken to complete 50 licks during CS presentation). Fig. 1B shows the level of conditioning to the noise CS expressed as the number of licks made during the first minute of CS presentation. The shaded histograms show how rats’ responses were moderated by the presence (dark grey) or absence (light grey) of the intermittent light distractor. Fig. 1C shows the suppression ratios upon test presentation of the light distractor. Fig. 1D shows the level of suppression to the light expressed as the number of licks made during the first minute of presentation. The shaded histograms show how rats’ responses depended on prior conditioning (dark grey) or were unconditioned (light grey). Error bars show two standard errors of the mean for approximate between groups comparisons.

There was a clear effect of trace on both suppression ratios to the noise CS, F(1,43) = 8.573, p = 0.005, as well as on the measure of learning provided by the number of licks made in the first 1 min of test, F(1,43) = 8.160, p = 0.007. Fig. 1 panels A and B show that, as expected, rats conditioned that the 0 s trace interval were more suppressed than those conditioned at the 10 s trace interval. Counter to prediction, there was no evidence that the light distractor moderated conditioning over the trace interval in that the interaction between trace and distractor was not significant for either measure, maximum F(1,43) = 0.004, p = 0.949 (Fig. 1A). Confirming that the light distractor was not without effect, there was a main effect on the suppression ratio measure of conditioning to the noise CS, F(1,43) = 4.786, p = 0.034 (Fig. 1A). This arose because rats presented with the flashing light distractor during conditioning were overall more suppressed than those conditioned without the ‘distractor’. Although non-significant, the same pattern was reflected in the licks measure of conditioned suppression (Fig. 1B).

Direct tests of conditioning to the light distractor stimulus showed no evidence of conditioning to this stimulus in that there was no difference in suppression ratios for rats conditioned with or without the intermittent light presented as a distractor, F(1,43) = 1.131, p = 0.294. Fig. 1C shows that the means were very comparable (overall SR = 0.378 with and 0.340 without). Given the lack of difference in suppression for rats conditioned with and without the distractor, the fact that the observed suppression ratios were below 0.5 can be attributed to unconditioned suppression and confirms the salience of the light distractor stimulus. The same pattern was reflected in the licks measure of conditioned suppression, only the interaction approached significance for the min 1 licks measure, F(1,43) = 3.758, p = 0.059 (Fig. 1D). However, ANOVA restricted to those rats actually presented with the light distractor at conditioning confirmed that there was also no effect of the trace interval at which the noise CS had been presented on either measure of conditioning to the light, maximum F(1,21) = 0.166, p = 0.688.

### 3.2 Experiment 2: light CS with intermittent noise distractor

**Fig. 2:**
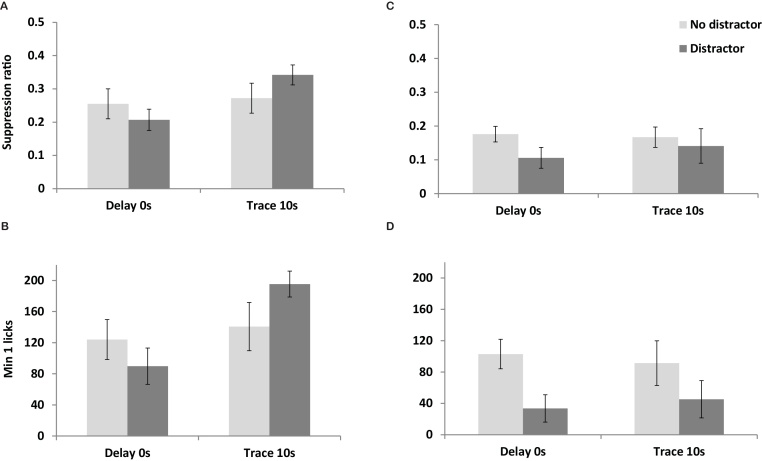
shows suppression measures of conditioning to the light CS and noise distractor in Experiment 2. Fig. 2A shows the level of conditioning to the light CS expressed as mean suppression ratio (calculated as A/(A + B); where A was the time taken to complete 50 licks prior to CS presentation and B was the time taken to complete 50 licks during CS presentation). Fig. 1B shows the level of conditioning to the light CS expressed as the number of licks made during the first minute of CS presentation. The shaded histograms show how rats’ responses were moderated by the presence (dark grey) or absence (light grey) of the intermittent noise distractor. Fig. 1C shows the suppression ratios upon test presentation of the noise distractor. Fig. 1D shows the level of suppression to the noise expressed as the number of licks made during the first minute of presentation. The shaded histograms show how rats’ responses depended on prior conditioning (dark grey) or were unconditioned (light grey). Error bars show two standard errors of the mean for approximate between groups comparisons.

The effect of trace on the suppression ratios to the light was marginal, F(1,44) = 3.824, p = 0.057 (Fig. 2A), but there was a clear effect of trace on the number of licks made in the first 1 min test presentation, F(1,44) = 6.116, p = 0.017 (Fig. 2B). There was no significant effect of the presence or absence of the intermittent noise distractor on conditioning to the light CS, maximum F(1,44) = 3.246, p = 0.078, for the interaction between trace and distractor for the 1 min drinking at test.

Direct tests of conditioning to the noise distractor showed no effect, F(1,44) = 1.836, p = 0.182, for the suppression ratio measure (SR = 0.123 with prior conditioning and SR = 0.171 without any prior conditioning; Fig. 2C). The fact that the observed suppression ratios were relatively low irrespective of whether the distractor had been present at conditioning means that its presentation resulted in unconditioned suppression and suggests that the intermittent noise stimulus was likely particularly salient. Although the suppression ratio measure was insensitive to its effects, rats conditioned in the presence of the noise distractor showed reduced drinking during the first 1 min test presentation of same the intermittent noise stimulus, F(1,44) = 6.539, p = 0.014 (Fig. 2D). ANOVA restricted to those rats actually presented with the noise distractor at conditioning confirmed that there was, however, no effect of the trace interval at which the light CS had been presented on either measure of conditioning to the noise, maximum F(1,22) = 0.343, p = 0.564.

### 3.3 Comparison of experiments 1 and 2

ANOVAs of the combined dataset confirmed the above conclusions in that there was a clear overall effect of trace interval on both the SR measure of conditioning to the target CS, F(1,87) = 10.927, p <0.001, as well as for 1st min licks, F(1,87) = 14.019, p < 0.001. There was no effect of distractor on these measures of conditioning to target, either overall or in interaction with trace, maximum F(1,87) = 1.716, p = 0.194. There was a main effect of CS identity on the suppression ratio measure of learning, F(1,87) = 23.243, p < 0.001, as well as for 1st min licks, F(1,87) = 11.561, p = 0.001, but no indication of any effects of target by trace interval, Fs <1. The only other significant effect was reduced licking to the noise as compared to the light distractor stimulus, F(1,87) = 6.016, p = 0.016, for 1st min licks.

## 4 Discussion

As would be expected, in both experiments, the introduction of a 10 s trace interval weakened associative learning compared with that seen in a 0 s conditioned group. However, there was no evidence of distraction in the groups conditioned in the presence of either an intermittent light (in Experiment 1) or an intermittent noise (in Experiment 2). On the contrary, in Experiment 1, associative learning to the noise was stronger (and for both trace and delay groups) for rats conditioned also in the presence of the intermittent light. As might be expected given that the intermittent light increased rather than decreased conditioning to the noise, there was no evidence to suggest that the light acquired any associative strength of its own.

Although based on a rat fear conditioning procedure, the procedure adopted in Experiment 1 was broadly similar to that used in the study of Han et al. (2003). Of course there may be species differences between rats and mice which can account for these discrepant findings and (relatedly) the conditioning parameters used for mice are quite different (see below).

One possibility which is important to consider is that of the modalities of the stimuli in use. Following the published Han et al. (2003) design, stimulus identity was not counterbalanced within Experiment 1: the noise was the CS and the intermittent light the notional distractor for all animals. Thus, taken in isolation, the results of Experiment 1 leave open the possibility that interference with trace conditioning might depend on stimulus modality and/or the level of arousal generated. In appetitive rat conditioning procedures, effects of stimulus modality have previously been found to outweigh differences due to the information value of the cue in relation to drug effects (Horsley and Cassaday, 2008). Moreover, in fear conditioning procedures, effects of stimulus modality have been found to depend on strain of rat (Norman and Cassaday, 2004). Therefore, in Experiment 2 of the present study, the stimulus roles were reversed: an intermittent light was the CS and the same noise stimulus was now presented intermittently in the background, to provide the putative distractor. In Experiment 2, when the roles of the stimuli were reversed, there was no effect of generally increased conditioning to the CS in the distractor condition. This lack of effect in Experiment 2 is consistent with the potential importance of stimulus modality. Moreover, combined analyses of Experiments 1 and 2, confirmed that the identity of the stimulus selected as CS was the major determinant of the level of associative learning, for both trace and delay conditioned groups. Nonetheless, the results of Experiment 2 do demonstrate the particular salience of the intermittent noise stimulus. Thus, the effect of modality could in effect be mediated by salience.

There was also a potentially important amodal difference between the stimuli which was likely to contribute to differences in perceived salience. The auditory CS used in Experiment 1 was continuous (as per Han et al., 2003; and the trace conditioning procedures routinely used in our laboratory). However, continuous light stimuli seem to be of relatively low salience and it is in any case difficult to match on the basis of physical intensity (loudness versus brightness) across modalities. We have typically used intermittent rather than continuous light CSs in an attempt to match the level of conditioning produced by alternative auditory versus light CSs as far as possible, and an intermittent light CS was used in Experiment 2. This was presented with the flashing parameters used previously to produce conditioning rather than those adopted when the flashing light was intended as a distractor in Experiment 1. We submit that the observed differences in conditioning with the auditory versus light CSs could also be attributable to differences in the amodal characterstics provided by their temporal differences. However, the difference in temporal parameters does not seem to provide the best account of the presence (in Experiment 1) versus absence of potentiation (in Experiment 2) as there should have been greater potential for within compound associations based on similarity in Experiment 2 (Rescorla and Gillan, 1980).

The discrepancy between the present result and the mouse fear trace conditioning variant still stands in need of explanation. It must be acknowledged that we did not attempt a full replication of the fear conditioning parameters used by Han et al. (2003) in mice. Instead we adapted a procedure developed specifically for rats and which has already been used in a number of trace fear conditioning studies, in an attempt to establish a reliable distractor suitable for use in rats. In the study conducted by Han et al. (2003), the response measure was freezing rather than conditioned suppression of a motivated response (as here). Accordingly, differences in the basic fear conditioning procedure in use included the duration of the pre-experimental lick training, the length of the CS, tone frequency and intensity, the number of conditioning trials and the shock intensity.

There were also two differences in the distraction aspect of the procedure. First, in order to make its temporal properties more dissimilar from our standard flashing light CS (500 ms on/off) which has proven an effective conditioning stimulus in the rat trace conditioning procedure, we increased the frequency with which the intermittent distractor was presented (130 ms on/off for 3 s durations, compared with 250 ms on/off for 3 s durations in Han et al., 2003). We cannot exclude the possibility that a distractor with temporal properties more similar to those used in the Han et al. (2003) would have had the desired effect of distracting from rather than enhancing learning about the trace-conditioned CS. The second difference can be seen as a strength in that, in Experiment 2, we also compared the effects of an intermittent noise stimulus as a distractor from the standard flashing light CS (500 ms on/off) in an otherwise identical procedure. This is an important comparison because even within species, effects of stimulus modality can depend on strain (Norman and Cassaday, 2004).The intermittent noise stimulus was similarly ineffective as a distractor, though there was no evidence for potentiation either in Experiment 2.

Potentiation effects of the kind demonstrated in the present study are not new. Indeed, a series of studies of autoshaping in the pigeon similarly showed that the deficit in conditioning otherwise produced by the introduction of a trace interval can be reduced by the interposition of another stimulus to fill the interval (Rescorla, 1982). Experimental analysis of this effect suggested that the observed potentiation was attributable to a ‘catalytic’ function of the interpolated stimulus, perhaps mediated by enhanced CS salience and encouraged by serial rather than concurrent presentation of the additional stimulus with the CS (Rescorla, 1982). Specifically, the potentiation effect was strongest when the interpolated stimulus was presented early in the trace interval. Moreover, studies of trace fear conditioning in the rat have demonstrated both distractor and potentiation effects depending on the duration of the interpolated stimulus (Hall and Honey, 1993). Specifically, longer cues which filled the trace interval provided more effective distractors, most likely because they were more likely to enter into association with the US and thus overshadow the CS (Hall and Honey, 1993). Interestingly, in the Han et al. (2003) study which relied on the role of the additional stimulus as a distractor, there was no evidence for overshadowing. It was not our objective to add to the literature with further exploration of the boundary conditions under which potentiation versus distraction is demonstrated, rather to report that parameters previously found suitable to set-up a stimulus as a distractor unexpectedly resulted in potentiation. In the present study (as per Han et al., 2003) the potential for an associatively-mediated effect – whether overshadowing, within-compound associations with the CS or direct associations with the US – was reduced by the intermittent presentation of the distractor.

### 4.1 Conclusions

Overall the results suggest that the presentation of an additional cue during the conditioning session can paradoxically improve conditioning to the target CS, depending on the relative salience of the additional cue. In principle, this finding could reflect potentiation based on within-compound associations (Durlach and Rescorla, 1980, Pearce et al., 1981). However, potentiation was seen only in Experiment 1 which used a continuous noise CS which was most dissimilar to the intermittent distractor (Rescorla and Gillan, 1980). Moreover, there was no evidence that either the light or noise distractor acquired associative strength and neither was there any difference between trace and delay conditioned groups in Experiment 1 in which the light distractor increased associative leaning to the noise. The finding that the presence of salient background cues can increase associative learning is consistent with arousal-mediated interpretations. However, the noise distractor which was more salient than the light had no such effect. Thus the level of arousal may be critical (Yerkes and Dodson, 1908).
